# Comparison of single- and triple-port VATS for lung cancer: A meta-analysis

**DOI:** 10.1515/med-2021-0333

**Published:** 2021-08-25

**Authors:** Yunfei Gao, Abulaiti Abulimiti, Dan He, Anpeng Ran, Dongbo Luo

**Affiliations:** Department of Thoracic Surgery, Affiliated Tumor Hospital, Xinjiang Medical University, Urumqi 830011, China

**Keywords:** VATS, single-port, triple-port, lung cancer, meta-analysis

## Abstract

**Objective:**

To compare the perioperative parameters between single- and triple-port video-assisted thoracoscopic surgery (VATS) lobectomy in the treatment of lung cancer.

**Methods:**

The Pubmed, Embase, Cochrane library, and the Web of Science databases were electronically searched from inception to September 2019 for all relevant studies. Study quality was evaluated using the Jadad scale or the Newcastle-Ottawa scale. The results were pooled using the generic inverse-variance method and expressed as mean differences or risk ratios, with 95% confidence intervals.

**Results:**

Three randomized controlled trials (RCTs) and ten cohort studies with 2,278 subjects were included in the meta-analysis. Whether based on RCTs or cohort studies, the pooled results showed no significant difference in the operation time, chest tube duration, intraoperative blood loss, postoperative hospital stays, lymph node dissection number, postoperative drainage volume, and postoperative complications between single- and triple-port VATS lobectomy (*P* > 0.05). Single-port VATS could relieve postoperative pain better than triple-port VATS, especially in the first day and fifth day (*P* < 0.05). No evidence of significant publication bias was found (*P* > 0.05).

**Conclusion:**

Single-port VATS lobectomy can yield similar perioperative results to those of triple-port VATS lobectomy and is more effective in relieving postoperative pain.

## 1 Introduction

Lung cancer is the most fatal cancer worldwide, which is characterized by uncontrolled cell growth in the lung tissues. It is estimated that 2.09 million new cases of lung cancer occurred globally in 2018, ranking first among all cancer types [1]. The etiology of lung cancer is not yet clear, and the myriad risk factors for lung cancer most commonly include lifestyle, environmental, and occupational exposures [2]. Although the survival rates for all cancers have improved in recent years, lung cancer survival remains at a relatively low level in China. In 2012–2015, the lung cancer survival rate in men and women were 16.8 and 25.1%, respectively [3]. Lung cancer poses a significant public health burden.

Lung cancer is broadly classified into two types, which grow and spread differently: small cell lung carcinoma and non-small cell lung carcinoma (NSCLC). Treatment options for lung cancer include surgery, radiation therapy, chemotherapy, and targeted therapy [4]. Although long-term survival remains poor for patients with metastasis, complete surgical resection is potentially curative for patients with early-stage lung cancer [5]. Video-assisted thoracoscopic surgery (VATS) is a type of thoracic surgery performed using a small video camera that is introduced into the patient’s chest via small incisions and has become a major surgical method in chest surgery [6]. Early in 2007, the American College of Chest Physicians’ evidence-based clinical practice guidelines for the treatment of stage I and II NSCLC consider VATS lobectomy to be an acceptable alternative to open thoracotomy [7]. Formerly, there was much debate about the feasibility of the technique in cancer surgery and proper lymph node handling [8]. Now, it is generally accepted that the outcome of a VATS procedure is at least not inferior to a resection via a traditional thoracotomy in the treatment of lung cancer [8].

VATS incision has many options, and the most frequently used option is one observation hole and 2–3 operation holes [9]. With the development of laparoscopic instrument technology, VATS is gradually reduced from multiple incisions to double incision, namely single utility port thoracoscopic surgery [10]. Early in 2011, Gonzalez-Rivas et al. reported their experience of single-port VATS lobectomy, the first worldwide-published study on major lung resection [11]. Single-port VATS has been developed in recent years, featured by minimal invasion and operation difficulty [12]. Both randomized controlled trial (RCT) and cohort study have reported that single-port VATS lobectomy showed similar results as triple-port VATS in safety and efficacy [13,14], indicating that single-port VATS lobectomy is a feasible and safe option for lung cancer patients. To the best of our knowledge, no systematic review has evaluated the curative effect between single-port and triple-port VATS for the surgical resection of lung cancer based on different study design. In this regard, the present systematic review aimed to compare the perioperative parameters between single- and triple-port VATS lobectomy for lung cancer treatment, so as to provide recommendation statement outlining VATS implication in lung cancer treatment.

## 2 Materials and methods

### 2.1 Literature search

This systematic review and meta-analysis followed the PRISMA statement and guidelines [15]. The literature research was performed using Pubmed, Embase, Cochrane library, and the Web of Science before September 2019. The search terms combined the following items: (“lung cancer” OR “lung carcinoma” OR “pulmonary cancer” OR “pulmonary carcinoma” OR “lung neoplasm” OR “pulmonary neoplasm”) AND (“thoracoscopic” OR “thoracoscopy” OR “thoracoscope”) AND (“Lobectomy” OR “pneumonectomy” OR “lung resection”). The search strategy applied a combination of title and abstract and used the Mesh Term (Table S1).

Reviewers were divided into two groups that worked in parallel. The reviewers independently screened each record by title, keywords, and abstract against the eligibility criteria. Full texts were referred to when information in the records was inadequate for determination. Any disagreement between the two groups of reviewers was resolved by an additional reviewer. Hand searching was performed by reviewing the references of included studies.

### 2.2 Selection criteria

The eligible studies included in this systematic review and meta-analysis met the following inclusion criteria: (1) patients with lung cancer; (2) different treatment groups adopted single-port or triple-port VATS lobectomy; (3) RCTs or cohort studies (retrospective and prospective); (4) the perioperative parameters were measured; (5) the language was restricted to English or Chinese.

The studies were excluded if: (1) case reports, conference abstracts, editorials, expert opinions, protocol, and commentaries; (2) lung cancer patients with other tumors; (3) studies with duplicate data reported in multiple studies by the same research group.

### 2.3 Data extraction

An extraction form was designed to extract data, including general information, methodological quality, clinical characteristics, and data of treatment outcomes. The data extraction procedure was also implemented independently by the two parallel groups of reviewers. Any disagreement was resolved by an additional reviewer.

### 2.4 Quality assessment

The quality of RCTs was evaluated using the Jadad scale, which was presented as a total scale of 1–5 based on assessment of randomization method, blinding, and descriptions of withdrawals and dropouts [16]. The quality of cohort studies was evaluated using the Newcastle-Ottawa scale (NOS) with the following domains: selection, comparability, and ascertainment of exposure/outcome [17].

### 2.5 Statistical analysis

Weighted mean differences (WMDs) in continuous variables and risk ratios (RRs) in dichotomous variables, with 95% confidence intervals (CIs) and *P* values, were calculated to assess effects of single- and triple-port VATS lobectomy for lung cancer. The inverse-variance method in continuous variables and Mantel-Haenszel method in dichotomous variables were used to combine data and generate the overall effect estimate. Heterogeneity was assessed by *Q* test and *I* ^2^ statistic, and *P* value of <0.10 or an *I* ^2^ value of >50% indicated substantial heterogeneity, thus determining the use of a fixed-effects model or random-effects model. Sensitivity analysis was performed based on cohort studies with propensity-matched analysis or not. Subgroup analysis was performed according to: (1) location (China, Korea), (2) sample size (≥200, <200), and (3) quality score (≥8, < 8). Meta-regression analysis was used to explore the potential source of heterogeneity. If more than 10 trials were included in the meta-analysis, publication bias was assessed using funnel plots and Egger’s test. Review Manager (RevMan) version 5.3 (The Nordic Cochrane Centre, Copenhagen, Denmark) was used for data syntheses. STATA version 15.1 (College Station, TX, USA) was used to analyze meta-regression analysis and publication bias.

## 3 Results

### 3.1 Characteristics of studies

**Figure 1:**
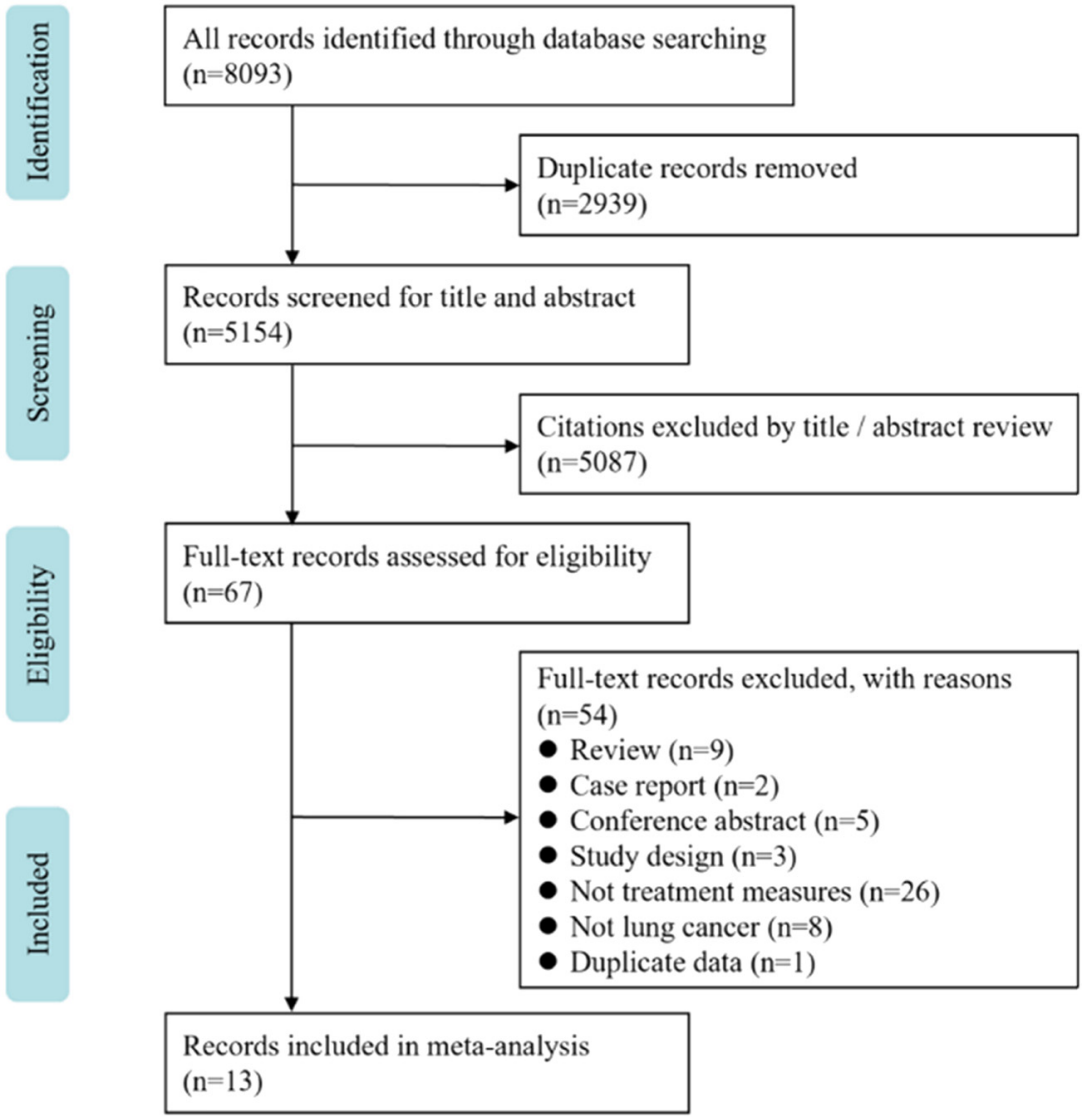
PRISMA flow chart for literature search.

As illustrated in Figure 1, our initial search identified 5,154 potentially references from four databases, from which 5,084 were excluded after screening the titles and abstracts. Ultimately, 13 studies were eligible for inclusion [13,14,18,19,20,21,22,23,24,25,26,27,28]. This selection consisted of 3 articles designed as RCTs [13,25,27] and 10 articles designed as cohort studies [14,18,19,20,21,22,23,24,26,28] (with 2 propensity-matched cohort studies [18,23]), with approximately 2,278 participants. Among the included studies, seven published in Chinese [19,21,22,24,25,26,27] and six in English [13,14,18,20,23,28]. Eleven studies were conducted in China [13,14,19,21,22,23,24,25,26,27,28], whereas others were performed in Korea [18,20]. There were four studies with sample sizes greater than 200 [19,20,21,26]. The characteristics of the articles are listed in Table 1.

**Table 1:** General information of all the included studies

Author, year	Type of study	Location	Study period	Groups	No. of cases	Age (years)^a^	Gender^b^	Tumor location^c^	Pathological types^d^	TNM stage^e^	Quality
Liu et al. 2018	RCT	China	2012/01–2016/01	Single-port	31	58.8 ± 13.7	25/6	11/20	8/21/2	13/14/4	4^*^
[25]				Triple-port	31	56.3 ± 11.9	22/9	12/17	8/21/2	14/12/5	
Shi et al. 2018	RCT	China	2017/02–2017/10	Single-port	80	61.77 ± 10.07	34/46	28/52	8/65/7	62/14/4	4^*^
[27]				Triple-port	96	60.04 ± 9.15	51/45	39/57	10/81/5	77/9/10	
Ye et al. 2019	RCT	China	2015/07–2017/01	Single-port	74	62.67 ± 9.16	43/31	24/50	10/59/5	59/15/0	5^*^
[13]				Triple-port	82	61.83 ± 8.26	59/23	29/53	14/61/7	64/18/0	
Han et al. 2017	Cohort study	Korea	2006/01–2015/06	Single-port	203	62.9(33–84)	132/71	79/124	46/113/8	120/33/14	8^**^
[20]				Triple-port	168	64.1(40–86)	105/63	73/95	49/86/19	89/33/23	
Hao et al. 2017	Cohort study	China	2015/03–2015/12	Single-port	208	59.2 ± 5.3	110/98	78/130	80/118/10	154/24/31	7^**^
[21]				Triple-port	103	59.7 ± 5.1	61/42	41/62	42/56./5	67/16/20	
Li et al. 2013	Cohort study	China	2011/02–2013/01	Single-port	87	63.86 ± 12.10	65/22	34/53	39/48/0	31/38/18	7^**^
[24]				Triple-port	75	66.20 ± 8.72	52/23	31/44	42/33/0	30/29/16	
Mu et al. 2015	Cohort study	China	2014/11–2015/05	Single-port	47	56.67 ± 11.62	25/22	18/29	2/45/0	27/9/2	9^**^
[23]				Triple-port	47	60.77 ± 11.04	14/33	20/30	5/42/0	26/5/1	
Rao et al. 2019	Cohort study	China	2017/08–2018/03	Single-port	153	56.1 ± 8.5	67/86	—	54/87/12	—	7^**^
[26]				Triple-port	102	54.4 ± 7.4	43/59	—	41/56/5	—	
Song et al. 2017	Cohort study	Korea	2011/12–2016/08	Single-port	26	64.8 ± 9.7	15/11	8/18	8/17/1	17/9/0	9^**^
[18]				Triple-port	26	65.0 ± 9.4	15/11	13/13	7/19/0	20/5/1	
Wang et al. 2017	Cohort study	China	2015/01–2015/12	Single-port	73	57.12 ± 6.43	31/42	—	16/34/23	27/21/3	9^**^
[14]				Triple-port	98	61.32 ± 7.54	53/45	—	13/52/33	34/30/7	
Wang 2018	Cohort study	China	2016/01–2017/08	Single-port	153	61.52 ± 9.70	82/71	57/96	26/115/12	115/14/24	7^**^
[19]				Triple-port	113	62.27 ± 10.08	69/44	47/66	25/81/7	72/10/31	
Xu et al. 2018	Cohort study	China	2017/09–2017/11	Single-port	60	61.4 ± 10.8	33/27	27/33	10/47/3	35/14/11	6^**^
[22]				Triple-port	60	63.5 ± 9.6	31/29	27/33	8/48/4	34/16/10	
Zhu et al. 2015	Cohort study	China	2014/08–2014/10	Single-port	33	62(25–79)	11/22	14/19	5/26/2	23/8/2	8^**^
[28]				Triple-port	49	59(31–81)	19/30	20/29	11/35/3	34/11/4	

Abbreviation: RCT, randomized controlled trial; TNM, tumor node metastasis.

^a^Values are presented as mean ± standard deviations or median (interquartile range); ^b^Male/Female; ^c^Right/Left; ^d^Squamous carcinoma/Adenocarcinoma/Others; ^e^stage I/stage II/stage III.

^*^Assessed using the Jadad scale; ^**^Assessed using NOS.

### 3.2 Quality of studies

Based on the Jadad scale, one study had a total score of 5 [13] and two studies had a total score of 4 (Table 1) [25,27]. The scores of the NOS quality assessment in cohort studies ranged from 6 to 9, four of them had scores greater than 8 [14,18,20,23,28]. All scores are listed in Table 1. Overall, most of the studies demonstrated a good or moderate methodology.

### 3.3 Perioperative parameters

**Figure 2:**
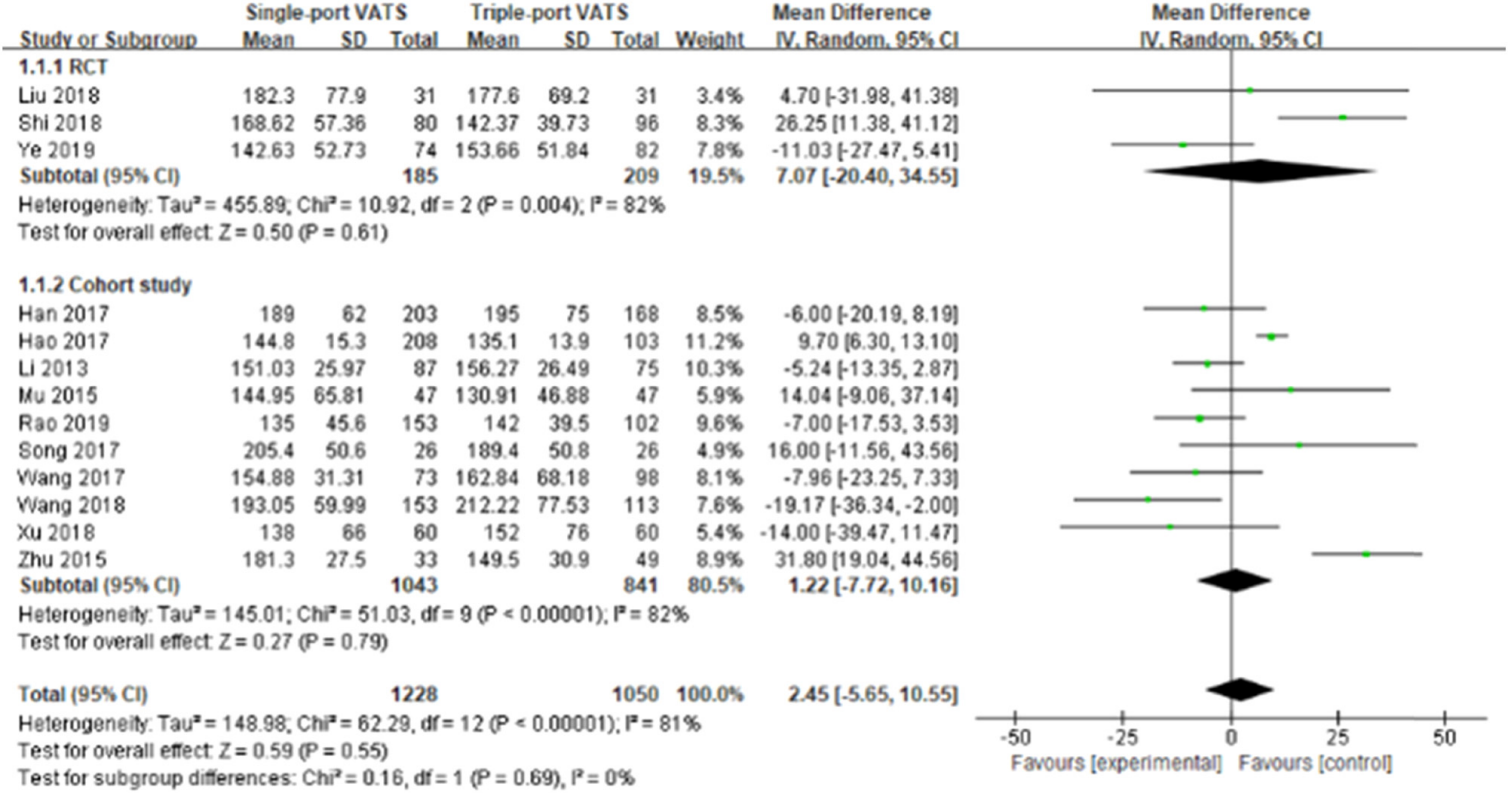
Forest plots of operation time for single- and triple-VATS lobectomy based on different research designs. For each study, the estimated OR (shown as square) and its 95% CI (shown as horizontal line) were plotted. The pooled OR and 95% CI were plotted as black diamond.

**Figure 3:**
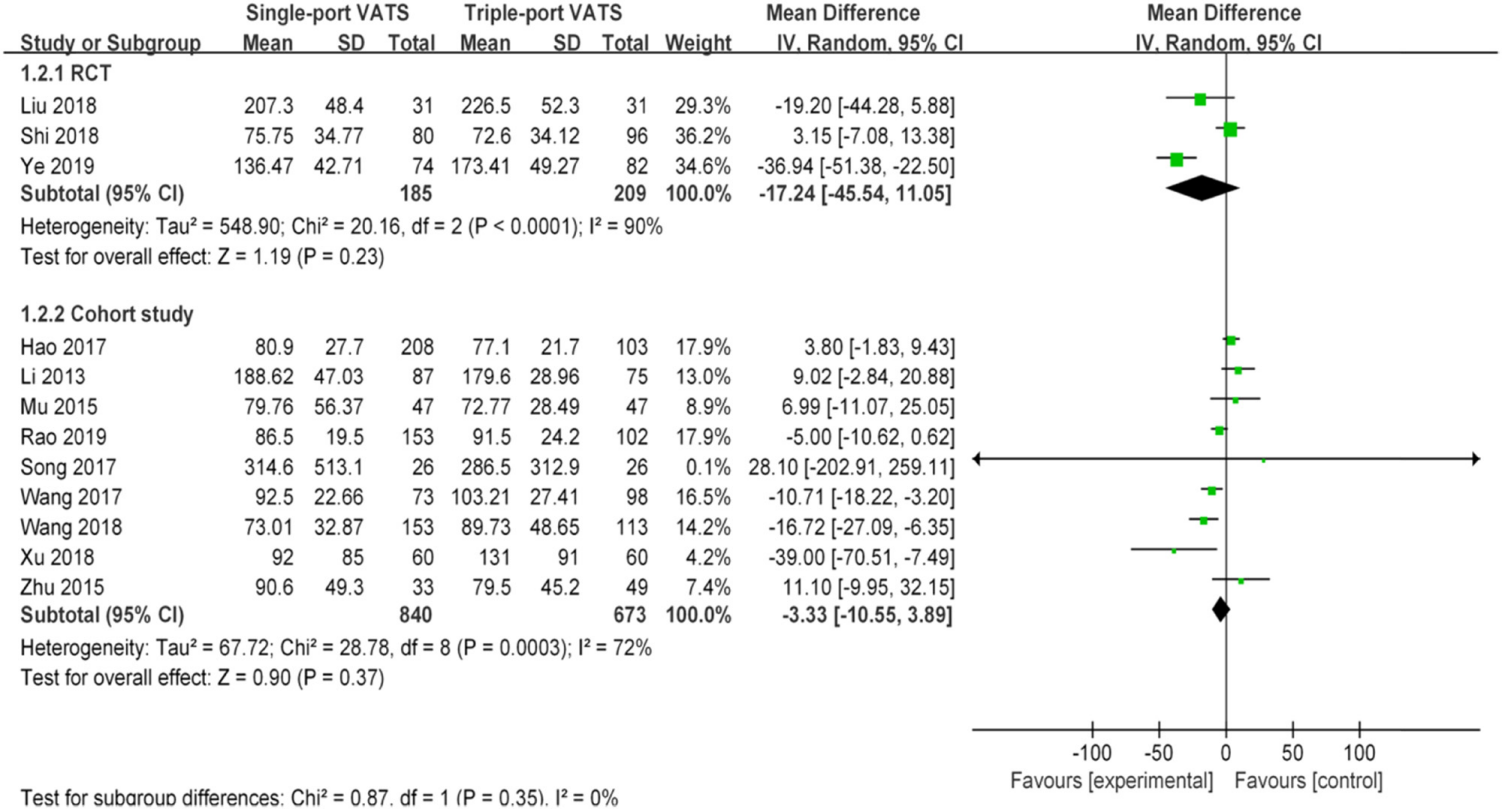
Forest plots of intraoperative blood loss for single- and triple-VATS lobectomy. For each study, the estimated OR (shown as square) and its 95% CI (shown as horizontal line) were plotted. The pooled OR and 95% CI were plotted as black diamond.

**Figure 4:**
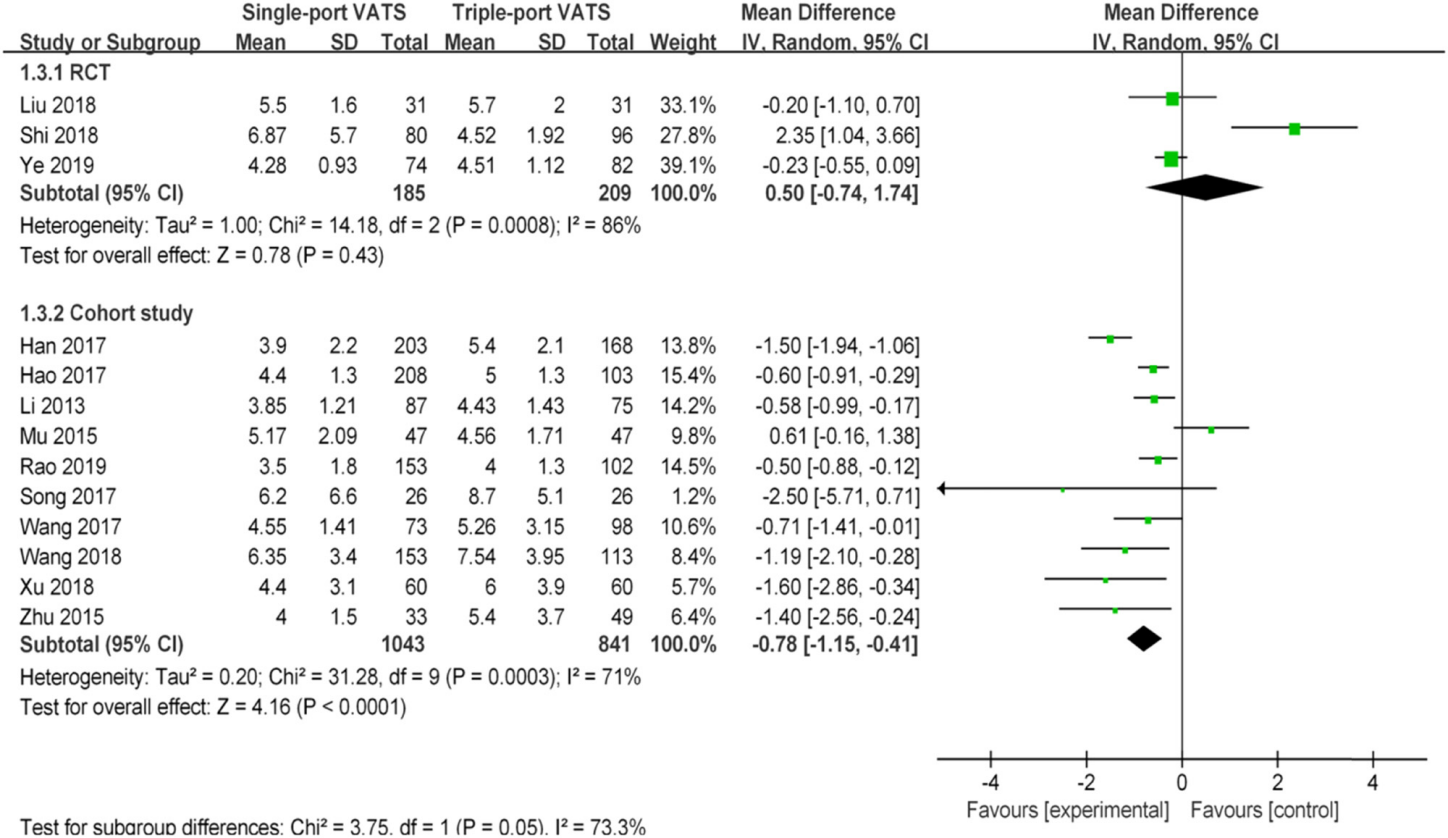
Forest plots of chest tube duration for single- and triple-VATS lobectomy. For each study, the estimated OR (shown as square) and its 95% CI (shown as horizontal line) were plotted. The pooled OR and 95% CI were plotted as black diamond.

**Figure 5:**
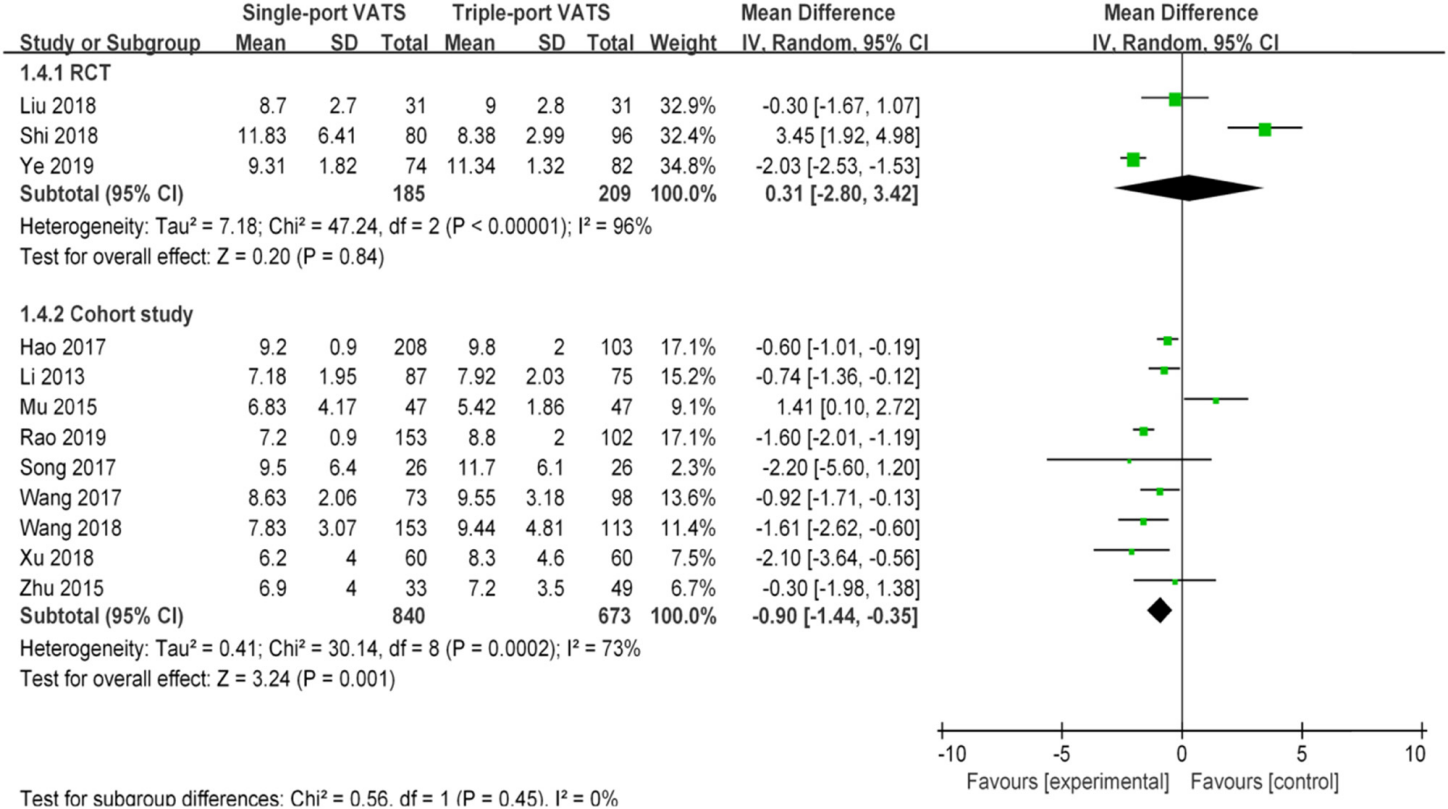
Forest plots of postoperative hospital stays for single- and triple-VATS lobectomy. For each study, the estimated OR (shown as square) and its 95% CI (shown as horizontal line) were plotted. The pooled OR and 95% CI were plotted as black diamond.

**Figure 6:**
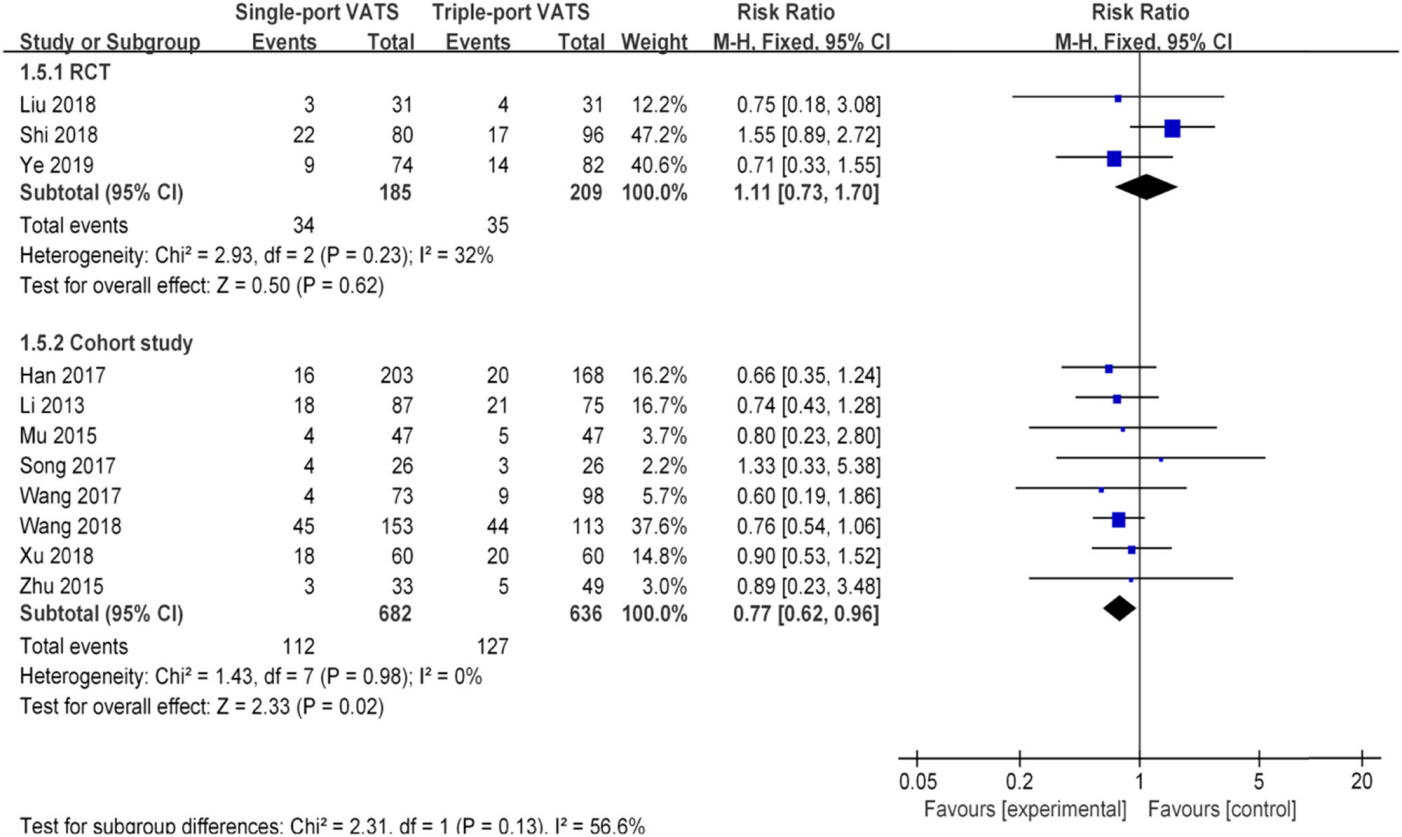
Forest plots of postoperative complications for single- and triple-VATS. For each study, the estimated OR (shown as square) and its 95% CI (shown as horizontal line) were plotted. The pooled OR and 95% CI were plotted as black diamond.

Nearly 20 perioperative parameters were reported in thirteen studies, of which the most frequently reported outcomes were operation time, chest tube duration, intraoperative blood loss, postoperative hospital stays, lymph node dissection number, postoperative pain score, postoperative drainage volume, postoperative complications, pulmonary leakage, pulmonary infection, atelectasis, arrhythmia, chylothorax, etc. Whether based on RCTs or cohort studies, the pooled results indicated that there was no significant difference in multiple perioperative parameters between single- and the triple-VATS lobectomy, except the other three outcomes (chest tube duration, postoperative hospital stays, and postoperative complications, Table 2, Figures 2–6). Based on the cohort studies and compared to the triple-VATS lobectomy, the single-VATS lobectomy showed shorter chest tube duration (days), shorter postoperative hospital stays (days), and lower risk of postoperative complications (WMD = −0.78, 95% CI: −1.15, −0.41, *P* < 0.01; WMD = −0.90, 95% CI: −1.44, −0.35, *P* < 0.01; RR = 0.77, 95% CI: 0.62, 0.96, *P* = 0.02, respectively; Table 2). However, combined results from RCTs showed that no significant difference in those outcomes was found between single- and the triple-VATS lobectomy (*P* > 0.05, Table 2). Results from combined RCTs and one cohort study showed that single-port VATS was better at reducing postoperative pain scores than triple-port VATS, especially in the first day and third day (first day for RCTs: WMD = −0.99, 95% CI: −1.25, −0.74, *P* < 0.01; first day for cohort: WMD = −0.28, 95% CI: −0.51, −0.05, *P* = 0.02; third day for RCTs: WMD = −0.90, 95% CI: −1.52, −0.28, *P* < 0.01, respectively; Table 2).

**Table 2:** Summary of perioperative outcomes between single- and the triple-VATS lobectomy based on different study designs

Perioperative parameters	RCTs					Cohort studies				
	Studies	Participants	MD (95% CI)	*P*	*I*^2^ (%)	Studies	Participants	MD (95% CI)	*P*	*I*^2^ (%)
Operation time (min)	3	394	7.07 (−20.40, 34.55)	0.61	82	10	1,884	1.22 (−7.72, 10.16)	0.79	82
Intraoperative blood loss (mL)	3	394	−17.24 (−45.54, 11.05)	0.23	90	9	1,513	−3.33 (−10.55, 3.89)	0.37	72
Chest tube duration (days)	3	394	0.50 (−0.74, 1.74)	0.43	86	10	1,884	−0.78 (−1.15, −0.41)	<0.01	71
						2^*^	146	−0.54 (−3.48, 2.40)	0.72	71
Postoperative hospital stays (days)	3	394	0.31 (−2.80, 3.42)	0.84	96	9	1,513	−0.90 (−1.44, −0.35)	<0.01	73
						2^*^	146	−0.04 (−3.51, 3.43)	0.98	74
Lymph node dissection number	2	332	−0.32 (−1.15, 0.50)	0.44	24	10	1,884	0.10 (−0.10, 0.29)	0.32	13
Postoperative drainage volume (mL)	1	156	−11.33 (−24.50, 1.84)	0.09	—	2	428	−147.21 (−339.25, 44.83)	0.13	78
Postoperative pain score (first day)	3	394	−0.99 (−1.25, −0.74)	<0.01	0	1	266	−0.28 (−0.51, −0.05)	0.02	—
Postoperative pain score (third day)	3	394	−0.90 (−1.52, −0.28)	<0.01	84	—	—	—	—	—

	Studies	Participants	RR (95% CI)	*P*	*I*^2^ (%)	Studies	Participants	RR (95% CI)	*P*	*I* ^2^
Postoperative complications	3	394	1.11 (0.73, 1.70)	0.62	32	8	1,318	0.77 (0.62, 0.96)	0.02	0
						1^*^	94	0.80 (0.23, 2.80)	0.73	—
Pulmonary leakage	2	332	1.17 (0.24, 5.70)	0.85	0	9	1,513	0.82 (0.55, 1.21)	0.31	0
Pulmonary infection	1	156	0.22 (0.01, 4.54)	0.33	—	8	1,351	0.76 (0.48, 1.19)	0.23	0
Atelectasis	2	332	0.77 (0.22, 2.67)	0.68	0	6	1,208	0.63 (0.37, 1.09)	0.10	0
Arrhythmia	2	332	0.76 (0.32, 1.79)	0.53	0	6	1,112	0.91 (0.57, 1.44)	0.68	0
Chylothorax	—	—	—	—	—	4	914	0.75 (0.21, 2.72)	0.66	0

Abbreviation: RCTs, randomized controlled trials; MD, mean difference; CI: confidence interval; RR, risk ratio.

^*^: Only included cohort studies with propensity-matched analysis.

### 3.4 Sensitivity analysis

Sensitivity analysis was performed on three outcomes with statistical differences between RCTs and cohort studies, namely, chest tube duration, postoperative hospital stays, and postoperative complications. When only propensity-matched cohort studies were included, the combined results showed no difference between the single- and triple-VATS lobectomy groups (chest tube duration: WMD = −0.54, 95% CI: −3.48, 2.40, *P* = 0.72; postoperative hospital stays: WMD = −0.04, 95% CI: −3.51, 3.43, *P* = 0.98; postoperative complications: RR = 0.80, 95% CI: 0.23, 2.80, *P* = 0.73; Table 2). The results were consistent with those of RCTs. These results showed that the pooled estimates were statistically significant in the sensitivity analysis.

### 3.5 Subgroup and meta-regression analysis

Subgroup and meta-regression analysis were performed to explore the potential source of heterogeneity in cohort studies where more than 10 studies were included. The results of subgroup analysis were summarized in Table 3. Further results from the univariate meta-regression analysis based on three grouping factors (location, sample size, and quality score) showed that there was no significant correlation between any of the covariates and the three perioperative outcomes (*P* > 0.05, Table 3).

**Table 3:** Subgroup and meta-regression analysis in cohort studies included

Perioperative parameters	Variables	Classification	Subgroup analysis					Meta-regression	
			Studies	Participants	MD (95% CI)	*P*	*I*^2^ (%)	*t*	*P*
Operation time (min)	Location	China	8	1,461	1.01 (−9.15, 11.16)	0.85	85	−0.13	0.902
		Korea	2	423	−1.39 (−14.01, 11.23)	0.83	48		
	Sample size	≥200	4	1,203	−4.23 (−17.78, 9.33)	0.54	86	−1.00	0.345
		<200	6	681	5.80 (−10.09, 21.70)	0.47	83		
	Quality score	≥8	5	770	9.22 (−8.76, 27.20)	0.31	82	1.50	0.172
		<8	5	1,114	−5.09 (−16.67, 6.49)	0.39	86		
Chest tube duration (days)	Location	China	8	1,461	−0.61 (−0.93, −0.30)	<0.01	55	1.90	0.093
		Korea	2	423	−1.52 (−1.95, −1.08)	<0.01	0		
	Sample size	≥200	4	1,203	−0.90 (−1.39, −0.41)	<0.01	79	−0.53	0.612
		<200	6	681	−0.69 (−1.33, −0.06)	0.03	67		
	Quality score	≥8	5	770	−0.86 (−1.80, 0.08)	0.07	83	−0.08	0.941
		<8	5	1,114	−0.62 (−0.82, −0.42)	<0.01	6		
Lymph node dissection number	Location	China	8	1,461	0.10 (−0.10, 0.29)	0.33	32	0.01	0.993
		Korea	2	423	0.06 (−1.81, 1.92)	0.95	0		
	Sample size	≥200	4	1,203	−0.00 (−0.22, 0.21)	0.99	38	−1.54	0.162
		<200	6	681	0.45 (0.04, 0.87)	0.03	0		
	Quality score	≥8	5	770	0.45 (−0.49, 1.39)	0.35	0	0.66	0.527
		<8	5	1,114	0.02 (−0.37, 0.42)	0.91	50		

Abbreviation: MD, mean difference; CI: confidence interval.

### 3.6 Publication bias analysis

Publication bias was examined only for three outcomes, namely, operation time, chest tube duration, and lymph node dissection number in cohort studies. The distribution of dots on the funnel plot was not significantly asymmetric (data not shown) and the results of Egger test were not significant (*P*  =  0.287, *P* = 0.483, and *P  =* 0.772 in operation time, chest tube duration, and lymph node dissection number, respectively), indicating the absence of publication bias in the present meta-analysis.

## 4 Discussion

This systematic review compared the perioperative parameters between single- and triple-port thoracoscopic lobectomy in lung cancer treatment. The combined results including 3 RCTs and 10 cohort studies showed that there was no statistical difference in the perioperative parameters of the two surgical methods, except postoperative pain score. Our results are consistent with the major findings of previous original studies with limited sample size [13,14,18,19,20,21,22,23,24,25,26,27,28]. Single-port VATS lobectomy is only performed in a limited number of hospitals in several countries due to its technical difficulty [18]. Our meta-analysis suggests that there was no obvious difference between single-port and triple-port thoracoscopic lobectomy, which laid the foundation for further promotion of single-port VATS lobectomy in the world.

Compared to conventional surgery, triple-port VATS thoracoscopic surgery has many advantages, such as less intraoperative blood lost, less pain, shorter duration of hospitalization, more rapid postoperative recovery [29], and long-term survival similar to that of conventional open surgery [30]. It is now generally accepted that the outcome of a VATS procedure is at least not inferior to a resection via a traditional thoracotomy [8]. Single-port VATS is a less invasive approach that allows major thoracic operations to be performed through a single small incision of about 4 cm, which can further reduce wounds and achieve the same effect as triple-port VATS thoracoscopic surgery [14]. Although our present study showed that there was no difference in postoperative indicators, combined results from RCTs and one cohort study both indicated that single-port VATS could relieve postoperative pain better than triple-port VATS, especially in the first day and fifth day. This may be one of the advantages of single-port VATS. Furthermore, the included RCTs also showed that single-port VATS could reduce trauma during surgery, reduce stress response, facilitate the recovery of postoperative quality of life, shorten the incision length, and improve scar appearance [13,27]. All these indicate that single-port VATS lobectomy is a feasible and safe option for lung cancer patients and should be popularized with its merits of minimal invasiveness.

The present meta-analysis included both RCTs and cohort studies with inconsistent results in chest tube duration, postoperative hospital stays, and postoperative complications. Although the results of well-designed observational studies (such as cohort study) do not systematically overestimate the magnitude of the effects of treatment as compared with those in RCTs, confounding is still a typical hazard of observational clinical research [31,32]. Propensity score methodology is a common approach to control confounding in nonexperimental studies of treatment effects via matching, stratification, regression adjustment, or any combination of these strategies [33]. When only propensity-matched cohort studies were included, there was no difference between the combined results from the RCTs and those from the cohort studies, further indicating that no difference was found in perioperative parameters between single-port and triple-port thoracoscopic lobectomy for lung cancer treatment. Nevertheless, when cohort studies are conducted in the future, various potential confounding biases should be controlled as much as possible.

There were several limitations of this systematic review and meta-analysis, as follows: (1) This study only analyzed perioperative parameters and failed to analyze long-term efficacy parameters. Even if we did not use the outcomes as a search term, few literatures with long-term outcome were found in literature screening. Future studies should clarify these questions when including single-VATS as a standard in thoracic procedures [20]. (2) Although the necessary data were not available, subgroup and meta-regression analyses were performed in three factors to explore the source of heterogeneity in cohort studies. We did not find any valuable heterogeneity factors. More extensive exploration may be needed, especially the differences in lung cancer subtypes. (3) Although extensive search strategies were used in present study, only Korean and Chinese studies were included at last. On the one hand, it may be related to the incomplete search of the database; on the other hand, it may be related to the epidemiologic distribution of lung cancer. Because China is one of the countries with a high incidence of lung cancer [3], there are a large number of cases for clinical study. We did not search the Chinese database because the quality of articles in Chinese journals has aroused concern in Chinese society [34]. Moreover, the methodological quality of those articles is often poor. There is also a selective publication bias in favor of positive results [35,36].

This systematic review and meta-analysis also showed some significant advantages. Most importantly, compared with published systematic review and meta-analysis, literature retrieval in this study was more comprehensive, and both RCTs and cohort studies were included with larger number of literatures. Therefore, the evidence was more reliable and scientific. Second, more adequate outcome indicators were analyzed in this meta-analysis. Thus, the stability of our research results was demonstrated. Finally, this systematic review and meta-analysis were performed following the PRISMA statement strictly, and the content was comprehensive.

In conclusion, the present systematic review and meta-analysis review comprehensively compare the perioperative parameters between single-port and triple-port thoracoscopic lobectomy for lung cancer treatment. Based on the combined results, single-port VATS lobectomy is a feasible option for lobectomy in lung cancer and may yield similar perioperative results to those of triple-port VATS lobectomy, and the former is more effective in relieving postoperative pain.
